# Simulation Guided Hand-Driven Portable Triboelectric Nanogenerator: Design, Optimisation, and Evaluation

**DOI:** 10.3390/mi12080955

**Published:** 2021-08-12

**Authors:** Yunzhong Wang, Anh Tran Tam Pham, Damian Tohl, Youhong Tang

**Affiliations:** Institute for Nanoscale Science and Technology, Medical Device Research Institute, College of Science and Engineering, Flinders University, Tonsley 5042, Australia; yunzhong.wang@flinders.edu.au (Y.W.); anh.pham@flinders.edu.au (A.T.T.P.); damian.tohl@flinders.edu.au (D.T.)

**Keywords:** hand driven, rotational triboelectric nanogenerator, simulation guidance, durability, capacitor charge

## Abstract

Inspired by the fundamental mechanics of an ancient whirligig (or buzzer toy; 3300 BC), a hand-driven rotational triboelectric nanogenerator (HDR-TENG) was designed and optimised, guided by our recently reported mathematical modelling. This modelling indicates that the power generated by HDR-TENG is a function of the number of segments, rotational speed, and tribo-surface spacing with different weighting sensitivities. Based on the simulation results, additive manufacturing technology was combined with commercially available components to cost-effectively fabricate the HDR-TENG. The fabricated HDR-TENG can provide stable and adjustable rotational speed up to 15,000 rpm with a linear hand stretching. The output voltage of HDR-TENG maintains a constant value within 50,000 cycles of testing when using Nylon 66 and PTFE as the triboelectric material. It can charge a 47 μF capacitor to 2.2 V in one minute. This study provides a cost-effective portable HDR-TENG device with adjustable high rotational speed, high power output, and long durable life, creating opportunities to provide a power supply for point-of-care devices in remote or resource-poor settings and applications in science and engineering education.

## 1. Introduction

The triboelectric nanogenerator (TENG) is one of the most popular methods to harvest electrical energy from mechanical energy such as vibration, sliding, rotation, etc. The main working modes of the triboelectric nanogenerator include the contact-separation mode and the sliding friction mode [1,2,3,4]. The contact-separation mode can significantly improve the durability life of the triboelectric material, but the output of the generator is relatively low compared to that of the sliding mode [5,6,7,8]. On the contrary, the sliding friction mode has greater output, but the triboelectric material’s lifespan is very short [9,10,11,12]. To improve the TENG’s workability, researchers have come to the strategy of the rotational TENG (R-TENG) [13,14,15,16]. This mechanism can not only provide adequate output but also lengthen the lifetime of the triboelectric material. Since the R-TENG operates in the grating-based sliding mode, its electrical output is advantageous for prolonged continuous energy harvesting.

R-TENG requires a starting force due to the friction and resistance forces that exist between the contact area of both triboelectric materials. For this reason, most existing R-TENG devices are driven by an AC/DC motor or some other external force such as wind energy or wave energy [17,18,19,20,21]. Using an AC/DC motor as the kinetic energy provider for R-TENG is a waste of energy because the AC/DC motor is driven by electricity. Alternatively, the forces provided by wind and ocean waves may not be enough to start R-TENG. Therefore, there exists the need for a rotational mode TENG, which provides adequate starting force and is highly portable. A recently reported hand-driven ultra-speed centrifuge easily operated by the linear reciprocating stretching and releasing of a wire attracted our attention [22,23]. This device has a low fabrication cost (20  cents), is lightweight (2 g), and can provide a maximum rotation speed of 12,800 rpm. It also provides a sufficient starting force while remaining portable and easy to operate. The combination of a manual hand-driven centrifuge and rotation TENG drives this research to innovate and apply hand-driven rational (HDR)-TENG [24]. We recently reported artificial intelligence enhanced mathematical modelling on R-TENG, considering the kinematic and geometric conditions [25] is used here to guide the HDR-TENG design. The optimal distributions for the charge, voltage, current, and harvested energy were all obtained from this simulation.

In this study, a high-speed HDR-TENG, optimised based on our mathematical modelling results, was fabricated, and evaluated with an easy operational method to convert human kinetic energy to electrical energy [26,27,28,29,30]. This HDR-TENG has significantly high durability, adjustable and stable rotation speeds, and the capability to charge a capacitor. The proposed HDR-TENG has applications for supplying power to point-of-care devices in remote or very remote or resource-poor settings and for science and engineering education.

## 2. Experimental

### 2.1. Device Fabrication and Assembling

These acrylic components in the HDR-TENG are cut by a laser cutter (Rayjet 300EDU, Australia), and the acrylic board has a thickness of 2 mm. The rotary disk and the stationary disk have a 130 mm diameter according to the suggested mathematic modelling [25]. The ball-bearing stopper has an inner diameter of 22 mm and an outer diameter of 31 mm. The other components are made by the 3D printing machine (Monoprice Maker Ultimate 2+, Rancho Cucamonga, CA, USA), and the material used is acrylonitrile butadiene styrene (ABS) plastic. The fasteners of the stationary and rotational parts have two holes with a diameter of 5 mm, which are used to place the driven wire for rotating the device. Meanwhile, another two smaller holes with a diameter of 1.5 mm inside the fasteners are used to host cables, which transmit the electrical energy from the rotational part to the output of the HDR-TENG. In this device, the stainless-steel ball-bearing E2.6003-2Z/C3 (SKF, Australia) is selected for its durability and stainproof ability. The bearing’s inner and outer diameters are 17 mm and 35 mm, respectively. This ball-bearing has a 3.25 N static load when using the stock grease, and we change the grease to a low-friction type (6003-2RS/C3, Spain) to decrease the driving force of the ball bearing to ensure that the HDR-TENG can easily rotate during the operation.

### 2.2. Triboelectric Material Selection

The triboelectric materials selected in this study were Nylon 66 and polytetrafluoroethylene (PTFE) with a thickness of 250 μm, which belong in opposite spectra of the triboelectric series [31,32]. They were cut into fan shape pieces by a laser cutter with a circle radius of 45 mm (Rayjet 300EDU, Sydney, Australia). The materials were then cleaned with ethanol to remove the residues from the surfaces. Both triboelectric materials have a 200 nm gold coating (Q300T-D, Quorum, UK) on the contact surface and 50 nm chromium on the electrode surface. The surface charge density after coating of Nylon and PTFE are 1150 kg/m^3^ and 2200 kg/m^3^, respectively [25].

### 2.3. Evaluation

**Figure 1:**
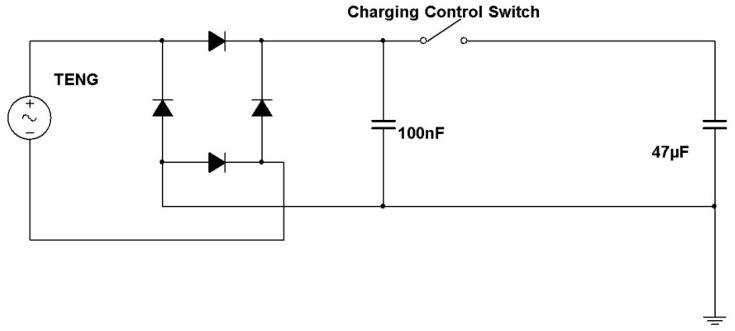
Circuit diagram of the full-bridge rectifier and charging circuit. The expected power supplier is the HDR-TENG device. The target charging component is the 47 µF capacitor.

The full-bridge rectifier used to convert AC voltage (generated by the HDR-TENG) to DC voltage is shown in Figure 1. The 100 nF capacitor is used to filter the high-frequency noise of the waveform to ensure that the waveform of DC voltage is as smooth as possible. The toggle switch is used to control the charging of the 47 μF capacitor used for energy storage.

## 3. Results and Discussion

### 3.1. Simulation Guided HDR-TENG Design

**Figure 2:**
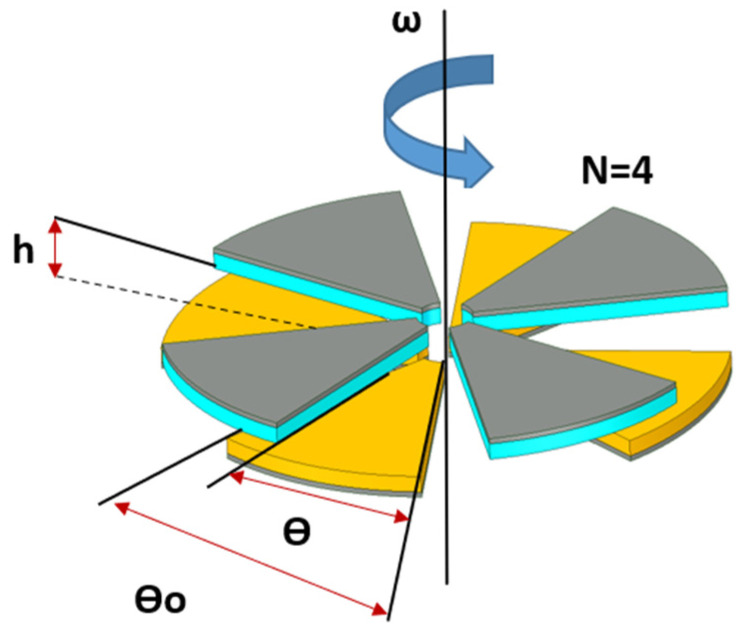
Schematic view of the rotational TENG, where *θ*_0_ and *θ* represent radians’ sector and rotation angles, respectively. *N*, *h*, and *ω* are the grating number, the gap between triboelectric material, and the rotational speed.

The schematic view of the rotational TENG is shown in Figure 2 with the geometry parameters.

From the previous simulation modelling [25], the short-circuit condition transferred charge (*Q_SC_*) is as follows:

(1) $$Q_{SC}=\frac{N\sigma \theta \left(\frac{d_{1}}{\varepsilon_{1}}+\frac{d_{2}}{\varepsilon_{2}}\right)\left(r_{2}^{2}-r_{1}^{2}\right)}{2\left(\frac{d_{1}}{\varepsilon_{1}}+\frac{d_{2}}{\varepsilon_{2}}+h\right)},\;\theta <\theta_{0}\;Q_{SC}=\frac{N\sigma \left(2\theta_{0}-\theta \right)\left(\frac{d_{1}}{\varepsilon_{1}}+\frac{d_{2}}{\varepsilon_{2}}\right)\left(r_{2}^{2}-r_{1}^{2}\right)}{2\left(\frac{d_{1}}{\varepsilon_{1}}+\frac{d_{2}}{\varepsilon_{2}}+h\right)},\;\theta_{0}<\theta <2\theta_{0}$$

Short-circuit current (*I_SC_*):

(2) $$I_{SC}=\frac{dQ_{sc}}{dt}=\frac{N\sigma \frac{d\theta }{dt}\left(\frac{d_{1}}{\varepsilon_{1}}+\frac{d_{2}}{\varepsilon_{2}}\right)\left(r_{2}^{2}-r_{1}^{2}\right)}{2\left(\frac{d_{1}}{\varepsilon_{1}}+\frac{d_{2}}{\varepsilon_{2}}+h\right)},\;\theta <\theta_{0}\;I_{SC}=\frac{dQ_{sc}}{dt}=\frac{N\sigma \frac{d\left(2\theta_{0}-\theta \right)}{dt}\left(\frac{d_{1}}{\varepsilon_{1}}+\frac{d_{2}}{\varepsilon_{2}}\right)\left(r_{2}^{2}-r_{1}^{2}\right)}{2\left(\frac{d_{1}}{\varepsilon_{1}}+\frac{d_{2}}{\varepsilon_{2}}+h\right)},\;\theta_{0}<\theta <2\theta_{0}$$

where $\omega =\frac{d\theta }{dt}$, *d*_1_ and *d*_2_ are the thickness of dielectric 1 and 2, respectively, $\varepsilon_{1}$ and $\varepsilon_{2}$ are the relative permittivity of dielectric 1 and dielectric 2, respectively. $\varepsilon_{0}$ is the vacuum permittivity, σ is the charge density, and $r_{1}$ and $r_{2}$ are constants for the inner radius and outer radius of the TENG, respectively. Based on the relationship in Equation (2), the value of the short-circuit current is dependent on h, which is the gap between the two triboelectric materials and the rotation speed, ω.

The capacitance, *C*, of the TENG can be calculated as follows:

(3) $$C=\frac{\varepsilon_{0}\frac{\left|\theta_{0}-\theta \right|\left(r_{2}^{2}-r_{1}^{2}\right)}{2}}{\frac{d_{1}}{\varepsilon_{1}}+\frac{d_{2}}{\varepsilon_{2}}+h}$$

The expression between open-circuit voltage, *V_oc_*, and the short-circuit condition transferred charge, *Q_sc_* is given as follows [14]:

(4) $$V_{OC}=\frac{Q_{SC}}{C}$$

If we assume *C* is a constant value and *Q_sc_* depends on *h,* which is the gap between the triboelectric material, as shown in Equation (2), the value of *V_oc_* will increase as *h* decreases. The relationship between output voltage, *V*, and the charge, *Q*, is shown in Equation (5) [14], where *C* is the capacitance of the TENG, and *Q* is the total charge.

(5) $$V=R\frac{dQ}{dt}=-\frac{1}{C}Q+V_{OC}$$

The main factor that affects the output voltage is the value of the load resistance, *R*, when *Q* and *C* are considered constants.

Our reported simulation modelling results [25] show that the resistance of the load is the major factor that will affect the output of the rotational mode TENG. Because the output power of the rotational mode TENG reaches the maximum value when the load resistance is in specific values. Other factors that will affect the output power are the gap between triboelectric material, the grating number, and rotational speed with decreased weightings. Based on the modelling results, a load resistance between 1 MΩ and 10 MΩ is the recommended range; a 10 MΩ load resistance has been selected for this study.

**Figure 3:**
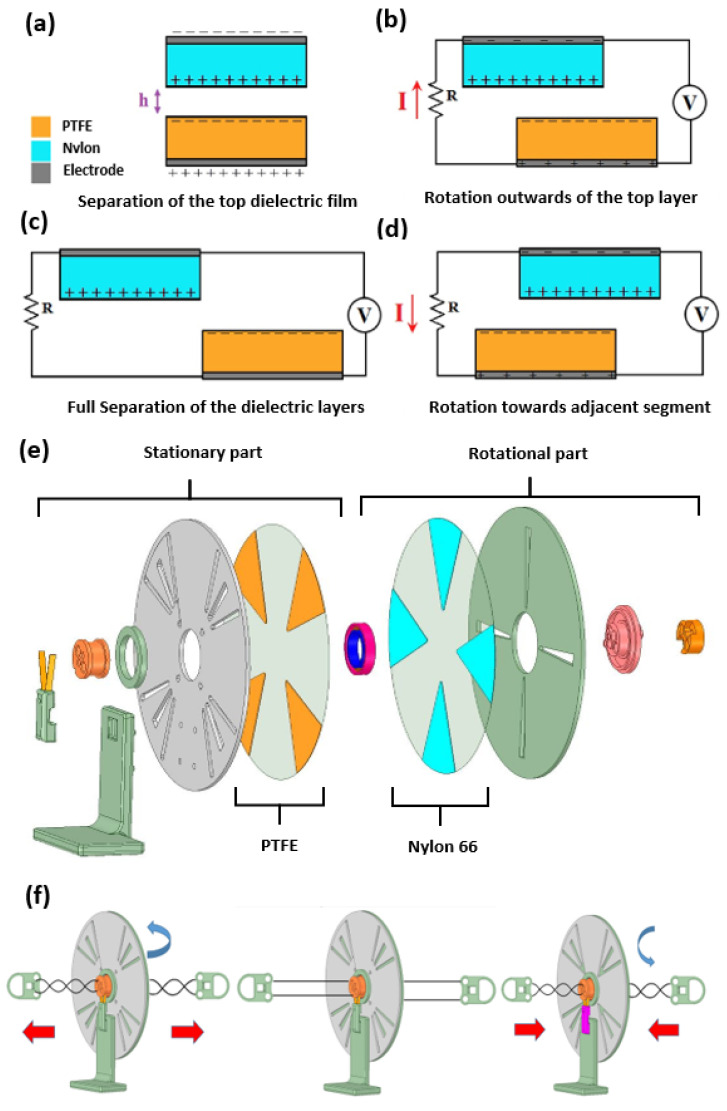
The working principle of the rotational TENG and the structure of the current HDR-TENG in this review; (**a**) two dielectric films are arranged so that there will be a controlled gap (h) between their surfaces; (**b**–**d**) the principle circuit applied on two dielectric films, When two dielectric films move relatively to each other, the change in the contact area generates the varying voltage in the circuit. Depending on the direction of the relative movements of two materials, the current in the circuit will have the corresponding direction; Reproduced with permission. [25]; (**e**) The explosion view of the HDR-TENG with all components divided into two groups of stationary part and rotational part. The orange and blue materials represent polytetrafluoroethylene (PTFE) and polyamide (Nylon 66). These two dielectric films, PTFE, and Nylon 66 are fixed on the two circle acrylic disks in grey and green. In the left-to-right order, the first component is the copper brush holder that is used to hold the copper brush (the orange V shape) that will collect the electrical energy from the Nylon 66; The L-shape support arm is used to position the whole device on a secured surface; The 2 orange and 1 pink pieces are used to hold two parts of the device together, as well as to collect the electrical energy from the rotational part (The green disk and the Nylon 66), then to transfer it to the copper brush component. The green ring is a limiting stopper used to hold the ball bearing. The grey disk with the PTFE dielectric film is the stationary part, which is fixed with the L-shape arm and will not rotate during the device’ operation. The groove on the stationary disk is designed to reduce the excess friction between two triboelectric materials, which will cause an unstable output waveform during the measurement. The pink and blue component is the high-speed low-friction ball bearing. The green disk with the Nylon 66 is the rotational part, which will rotate during the device operation; (**f**) the operation steps of the device; when stretching and releasing the wires, the rotating disk will accelerate from zero to its maximum rotation speed before decelerating to zero, then the rotating disk will reverse its rotation in the second period of stretching and releasing the wires.

For the mechanism of R-TENG, the gap distance between two triboelectric materials, *h*, is shown in Figure 3a. The cycle of energy generation is shown in Figure 3b–d. When the rotational part starts to rotate, a flow of the electrons transfers from the negative charge material (PTFE) to the positive charge material (Nylon 66) to keep the electric neutrality between two triboelectric materials, as shown in Figure 3b. These two triboelectric materials need full separation to achieve the maximum charge, as shown in Figure 3c. A flow of electrons is then transferred from the positive charge material (Nylon 66) toward the negative charge material (PTFE) so that a current with the opposite direction can be generated, as shown in Figure 3d.

The HDR-TENG consists of eight components, as shown in Figure 3e. The first component is the copper brush holder used to hold the copper brush to collect the electrical energy from the Nylon 66. The L-shape support arm is used to position the device on a secured surface. The green ring is a limiting stopper used to hold the ball bearing. The grey disk is the stationary disk used to hold the PTFE. The groove on the stationary disk is designed to reduce the excess friction between two triboelectric materials, which will cause an unstable output waveform during the measurement. The pink and blue component is the high-speed low-friction ball bearing, which provides stable high-speed rotation and is an interconnection shaft between the stationary and rotational parts. The green disk represents the rotating disk used to hold the Nylon 66. The last component is the rotational core that is used to adjust the gap between two triboelectric materials. This component also assembles the rotational part and stationary part as one system. To operate the HDR-TENG, the rotational disk is first manually rotating to store the initial driving force for the HDR-TENG, then hand-to-linear reciprocating is used to stretch and release the wire, then the rotational part will be driven by force to generate electrical power, as shown in Figure 3f.

### 3.2. Device Optimisation

**Figure 4:**
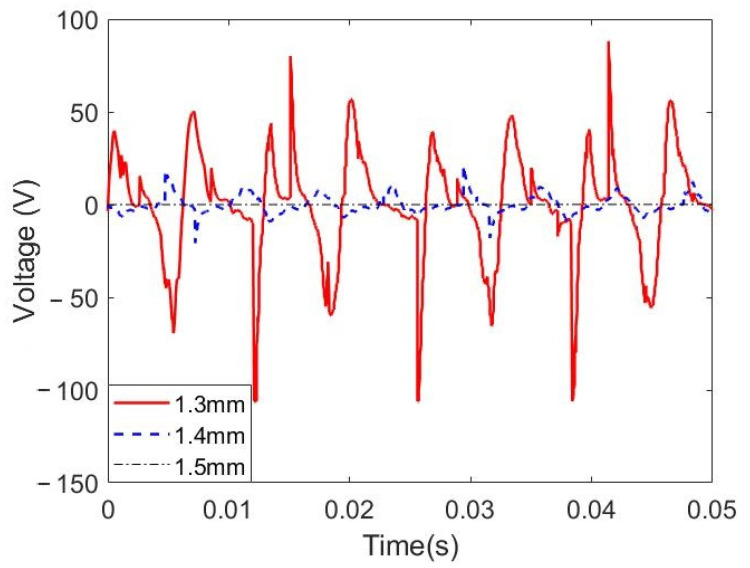
Optimization of the gap between rotational element and stationary element by measuring the open-circuit voltage, *V_oc_*, of the HDR-TENG. Before reaching the contact point (the gap between two dielectric materials is 1.2 mm), the output voltage of the device increases when the gap is reduced from 1.5 mm to 1.3 mm and reaches the maximum output voltage of 126.63 Vpp with a gap of 1.3 mm.

Based on the mathematical modelling simulation result, the distance between two materials is directly proportional to the output power [25], which means the closer together the triboelectric materials are, the more electrical energy will be generated. However, a gap that is too small will cause the triboelectric materials to contact each other due to the tolerance of the 3D printed components, which will significantly reduce the useful life of the triboelectric material. Therefore, the open-circuit voltage, *V_oc_*, of the HDR-TENG at the different gaps between stationary and rotational disks has been measured experimentally. When the gap between the two material films is larger than 1.5 mm, the triboelectric materials do not have a triboelectric effect, resulting in the HDR-TENG failing to work at the optimum operating condition. When the gap is smaller than 1.2 mm, the HDR-TENG cannot rotate freely due to the excessive friction between triboelectric materials produced in the rotation. Therefore, gaps of 1.3 mm, 1.4 mm, and 1.5 mm were chosen for further optimisation. The open-circuit voltage, *V_oc_*, results of the HDR-TENG for these gaps are shown in Figure 4. The HDR-TENG can only generate 5 Vpp voltage when the rotational and stationary elements gap is 1.5 mm. The output of the HDR-TENG increases approximately four times to 18.9 Vpp when the gap between the rotational element and stationary element is 1.4 mm. When the gap is reduced to 1.3 mm, the HDR-TENG can generate a voltage of 126.63 Vpp. Hence, the rotational core gap of 1.3 mm gap is the best choice for the HDR-TENG in this device.

The critical rotational speed, *φ_Critical_*, is given as follows [22]:

(6) $$\varphi_{Critical}=\frac{L\sqrt{\pi^{2}-2^{2}}}{2R_{s}}$$

where *R_s_* is the string radius and *L* is the string length.

The angular velocity is expressed as follows [22]:

(7) $$\omega =\sqrt{\frac{2F_{m}R_{s}{}^{2}}{IL}}$$

where, *F_m_* is the peak amplitude of the input force or the inertial moment of the rotation disc.

Then, the maximum rotational speed is as follows:

(8) $$\emptyset_{Max}=\emptyset_{Critical}\times \omega$$

By substituting Equations (6) and (7) into Equation (8), the expression of the maximum rotational speed is obtained as follows:

(9) $$\emptyset_{Max}=\frac{L\sqrt{\pi^{2}-2^{2}}}{2R_{s}}\times \sqrt{\frac{2F_{m}R_{s}{}^{2}}{IL}}=\sqrt{\frac{LF_{m}}{I}}$$

**Figure 5:**
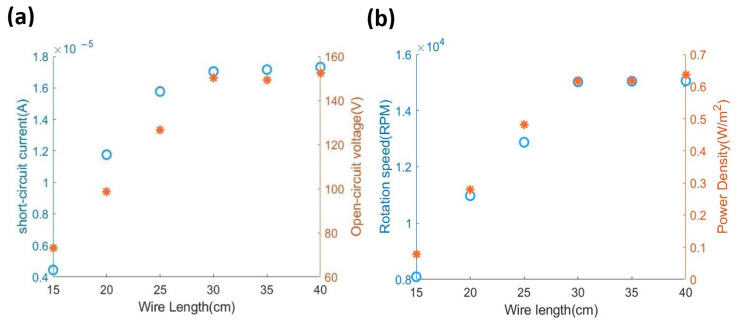
The result of HDR-TENG using different length cables. (**a**) Optimization of the wire length of the device. The blue circles represent the short-circuit current, and the open-circuit voltage is represented by the red stars. The load resistance for the test is 10 MΩ. (**b**) Rotational speed and power density at different wire lengths.

As reported in the mathematical modelling [25], the rotational speed is directly proportional to the output power. In the HDR-TENG, the rotational speed can be controlled by the wire length when the input force and inertial moment are considered constants for the certain system, as given by Equation (9). Different wire lengths (15 cm, 20 cm, 25 cm, 30 cm, 35 cm, and 40 cm) were used to measure the HDR-TENG’s open-circuit voltage, Voc, and short-circuit current results are shown in Figure 5. The open-circuit voltage of the HDR-TENG increases linearly with the length of wire, and the short-circuit current of the HDR-TENG starts to reach saturation when wire length is over 30 cm. The saturation condition of the output current means most electrons existing on the surface of the triboelectric material are fully utilized by this optimised device [33]. The saturation condition has rarely shown up on the hand-driven mode TENGs before, with much lower rotating speeds [24]. Thus, with a stable high-rotation speed, the proposed device can successfully reach the saturation condition of the tested materials.

From Figure 5a, it is clear the open-circuit voltage of HDR-TENG is directly proportional to the wire length. However, another factor requiring consideration in the design of the HDR-TENG is its ease of operation. Therefore, a wire length of 25 cm has been chosen to balance the voltage output and effective operation. Figure 5b indicates the relationship between cable length and the output power of HDR-TENG. Based on these results, the optimum performance of the HDR-TENG was achieved with a gap of 1.3 mm between the stationary disk and the rotational disk is 1.3 mm and a wire length of 25 cm, as in Figure 5b. These geometry and design parameters will be utilized in the HDR-TENG for its performance evaluation.

### 3.3. Performance Evaluation

**Figure 6:**
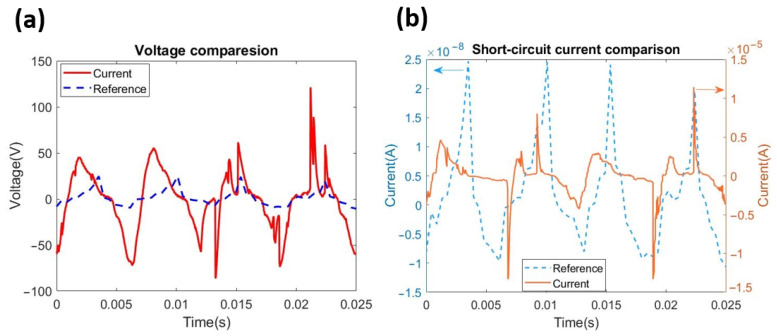
The results of the HDR-TENG and DC motor driven R-TENG for (**a**) the open-circuit voltage and (**b**) the short-circuit current. The dashed blue line represents the results of the DC motor-driven R-TENG used in the previous study [25], and the solid red line represents the measurement results of the proposed HDR-TENG.

The short-circuit current and the open-circuit voltage of the proposed HDR-TENG were measured with a maximum stabilised rotation speed of 12,800 rpm and compared with a 12 V DC motor-driven rotational mode TENG with a maximum stabilised rotation speed of 400 rpm [25]. The geometry and triboelectric material setups for both the HDR-TENG and the DC motor-driven R-TENG are the same. From Equation (2), the output current of the rotational mode TENG is dependent on the rotation speed. The rotation speed of the DC motor reaches a maximum of 420 rpm [25], while the proposed HDR-TENG device can achieve a speed of over 12,800 rpm, this dramatically improves the output of the TENG. The open-circuit voltage of the HDR-TENG is 126.63 Vpp, and the open-circuit voltage of the DC motor driven R-TENG is 32.95 Vpp, as shown in Figure 6a. The proposed HDR-TENG has an improvement in the output voltage of almost four times. The short-circuit current of the proposed HDR-TENG is 15.77 μA, while the DC motor-driven R-TENG can only produce 24.68 nA, as shown in Figure 6b. The proposed HDR-TENG shows an improvement in the short-circuit current of over 630 times. Based on this comparison, the hand-driven optimized design of the proposed HDR-TENG significantly shows improvement over the DC motor-driven R-TENG.

**Figure 7:**
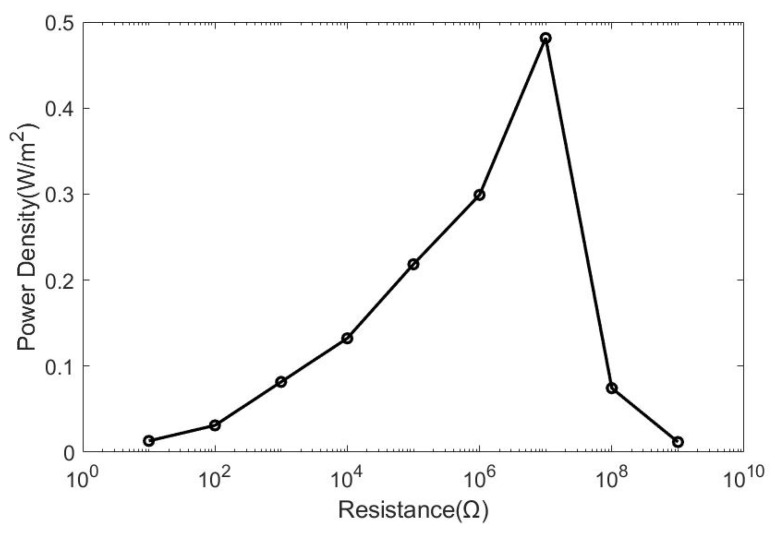
Power density of the HDR-TENG on the varying loading resistance. The specification of the HDR-TENG: *h* is 1.3 mm, the rotation speed is 12,800 rpm with a 25 cm wire length, and the load resistance is 10 MΩ.

Figure 7 shows the power density of HDR-TENG under different loads. It can be seen that the power density increases with resistance until the load resistance reaches 10 MΩ and the power density is 0.481 W/m^2^ in the experiment. The power density reduced significantly after when the load resistance increased from 10 MΩ to 1 GΩ. Therefore, the output power of the HDR-TENG will reach its maximum when utilizing a 10 MΩ resistor as load.

**Figure 8:**
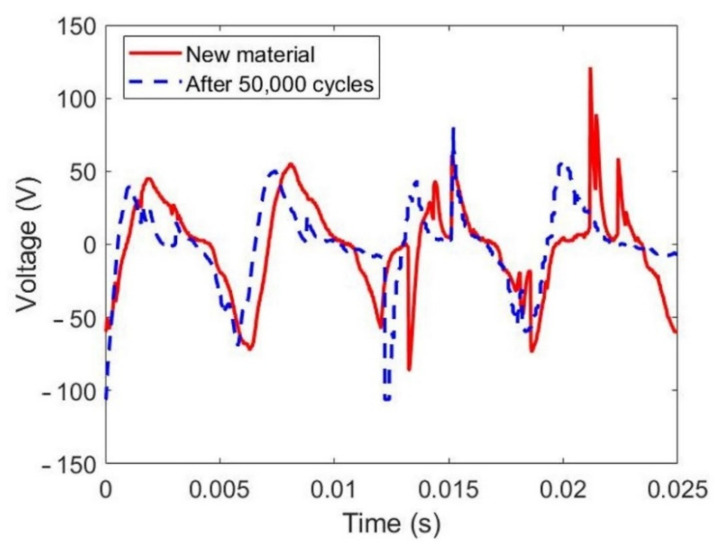
Durability evaluation of the output voltages of a new HDR-TENG compared to an HDR-TENG after 50,000 cycles of rotation. The specification of the HDR-TENG: *h* is 1.3 mm, the rotation speed is 12,800 rpm with a 25 cm wire length, and the load resistance is 10 MΩ.

The durability of the HDR-TENG has been evaluated, and the results are shown in Figure 8. The solid red line represents the output voltage of a new HDR-TENG, which can generate 126.63 V. The dashed blue line represents the output voltage of the HDR-TENG after 50,000 cycles of testing, which can generate 124.7 V. The proposed optimization of the HDR-TENG can efficiently improve the useful life of the triboelectric material, which is one of the major existing problems for rotational mode TENG.

**Figure 9:**
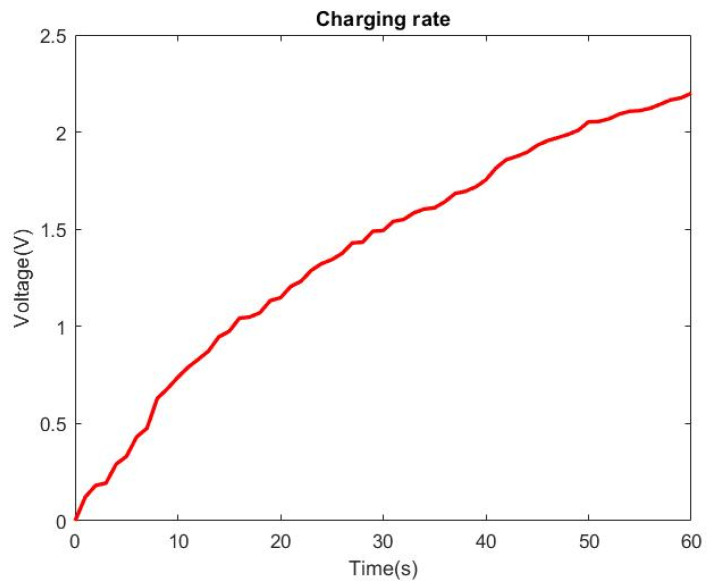
The charging curves of the 47 μF capacitor with the HDR-TENG. The specification of the HDR-TENG: *h* is 1.3 mm, the rotation speed is 12,800 rpm with a 25 cm wire length, and the load resistance is 10 MΩ.

To verify the output of the HDR-TENG can be utilized, it was connected to the rectifier and charging circuit shown in Figure 9 and used to charge a capacitor. A digital multimeter was connected to the end of the 47 μF capacitor to measure the charged voltage. Figure 9 shows that the 47 μF capacitor reaches 2.2 V after 60 s of charging using the HDR-TENG to supply power.

## 4. Conclusions

Guided by our previous reported simulation modelling, a cost-effective, controllable high-speed rotational hand-driven triboelectric nanogenerator has been proposed. The HDR-TENG enables continuous reciprocating stretching and releasing of a wire to obtain kinetic energy from the human body to actively control the performance of the rotational mode TENG. From our experimental results, the proposed HDR-TENG can utilize most electrons existing on the surface of the triboelectric material to generate current. The proposed HDR-TENG also works at an ultra-high rotational speed of over 12,800 rpm to provide a maximum output voltage of 126.63 V and a power density of 0.481 W/m^2^. The power generated by our proposed HDR-TENG can sustainably charge a 47 μF capacitor to 2.2 V in a minute and continues to have a stable output voltage after 50,000 cycles. The exchange charge density of the used triboelectric materials arising from the triboelectric charge exchange is 114.38 μC/m^2^. Based on the measurement result, the optimized device can efficiently utilize all the charges on the triboelectric material’s surface to generate electrical energy. In the future study, we will search for the material with more charges on the surface to enhance the output power of the HDR-TENG.
